# Deciphering Childhood Rosacea: A Comprehensive Review

**DOI:** 10.3390/jcm13041126

**Published:** 2024-02-16

**Authors:** Yu Ri Woo, Hei Sung Kim

**Affiliations:** Department of Dermatology, Incheon St. Mary’s Hospital, College of Medicine, The Catholic University of Korea, Seoul 21431, Republic of Korea; w1206@naver.com

**Keywords:** rosacea, children, pediatric, diagnosis, management

## Abstract

Childhood rosacea is a lesser known, yet significant, skin condition presenting diagnostic and treatment challenges. Although often underdiagnosed due to unclear diagnostic criteria, it manifests similarly to adult rosacea, with features such as papulopustular, telangiectasia, granulomatous, idiopathic facial aseptic granuloma, and ocular rosacea. The complex pathophysiology involves genetic, immunological, and environmental factors. Distinguishing childhood rosacea from conditions like acne, steroid rosacea, sarcoidosis, and lupus vulgaris is crucial but complicated by the lack of established criteria. Treatment strategies, mainly extrapolated from adult management protocols, include topical therapies, systemic medications, and laser treatments, adapted for pediatric patients. Special attention is given to ocular rosacea, often preceding skin manifestations, necessitating multidisciplinary care. The review underscores the urgent need for clear diagnostic guidelines, increased awareness, and tailored pediatric treatment protocols to improve patient outcomes and mitigate the condition’s evolution into adulthood.

## 1. Introduction

Rosacea is a chronic inflammatory skin disease that can affect the skin and eyes. The pathogenesis of rosacea is complex and related to interactions between genetic and environmental factors, the dysregulation of innate and acquired immune systems, the dysregulation of nerves and blood vessels, and the imbalance of the skin microbiota, particularly the overgrowth of Demodex mites. Although rosacea is generally commonly observed in adults, mainly in middle-aged women in their 30s to 50s [1], it can also be observed in children. However, clear diagnostic criteria for rosacea in children have not been established yet. Therefore, the epidemiology, clinical features, and updated treatment options of childhood rosacea are reviewed in detail.

## 2. Epidemiology

Currently, the exact prevalence and incidence of childhood rosacea are not well known. A recent study by Hoepfner et al. [1] reported that childhood rosacea was diagnosed in less than 1% of children in their single-center study. Another study conducted in Colombia reported that 1.4% of rosacea patients were younger than 20 years old [2]. In a study identifying the hospital visit tendency of rosacea patients, 1.2% of the rosacea visits were observed among rosacea patients in their 20s or younger [3]. An epidemiological study of rosacea conducted in the United Kingdom found that the incidence rate (IR) of rosacea in patients younger than 20 years old was 0.89 (95% confidence interval [CI]: 0.87–0.91) per 1000 person-years [4]. 

Regarding the age of onset, some studies have reported that childhood rosacea occurred at an average age of 4 to 5 years [5,6]. 

As for the sex, rosacea is most frequently observed in women in general [4,7]. A study by Spoendlin et al. [4] showed a slightly increased incidence rate in women (IR = 0.95; 95% CI: 0.92–0.98) compared to men (IR = 0.80; 95% CI: 0.80–0.86). In children, rosacea is similarly observed both in boys and girls [5,8]. Although childhood rosacea can occur in all phototypes, the papulopustular type is more frequently observed in relatively light phototypes, and granulomatous rosacea occurs equally in patients with both dark and light skin [8].

### 2.1. Pathophysiology

The pathogenesis of rosacea remains unclear. The interplay between genetic predisposition and environmental triggers, dysregulation of the immune system, imbalance of neurovascular system, and interactions with skin and gut microbiota are considered important factors in the pathogenesis of rosacea. 

A family history of rosacea has been reported in up to 30% of rosacea patients across all ages [9], indicating a genetic component in the pathogenesis of rosacea. A recent study reported that pediatric demodicosis with rosacea-type rash is associated with gain-of-function mutations in STAT1 [10]. A recent case series also reported that most early-onset rosacea patients showed the gain-of-function mutation in STAT1 [11], suggesting a genetic predisposition to rosacea in early-onset rosacea cases. 

As for the innate immune system, lesional skin of rosacea patients showed increased expression of Toll-like receptor 2 and matrix metalloproteinase, which induces an increase in cytokines and antimicrobial peptides, including cathelicidin. Rosacea patients showed increased expression of cathelicidin in their skin. An active form of cathelicidin, LL-37, induces the infiltrations of various inflammatory cells, angiogenesis, and cytokine releases. With regard to inflammation, the role played by Th17, secreted by T cells, macrophages, monocytes, and natural killer cells, has recently begun to emerge [12,13]. 

The dysregulation of nerves and blood vessels is also a very important factor in the pathophysiology of rosacea. Some rosacea triggers, such as ultraviolet radiation and temperature change, induce activation of transient receptor potential (TRP) cation channels, which are widely expressed in neurons, keratinocytes, and endothelial cells. This signaling induces the release of neurogenic mediators such as substance P and calcitonin-gene-realized peptides in rosacea. The release of neurogenic mediators is associated with persistent flushing experienced by patients and a lower threshold for heat and pain [8]. 

In addition, it is known that an imbalance in the skin microflora affects the etiology of rosacea. Several studies have reported a high density of Demodex in rosacea patients [14,15,16]. The overgrowth of Demodex can be perpetuated by blocking substances necessary for regulating the type 2 immune response by helper T cells [8]. Also, in patients with papulopustular rosacea, the overgrowth of β-hemolytic Staphylococcus is observed, which can lead to the activation of Toll-like receptor 2 and is known to lead to the associated immune dysregulation [8]. 

While inflammation, immune dysregulation, and neurovascular changes are fundamental to rosacea’s pathophysiology in both adults and children, specific genetic mutations are more closely associated with early-onset cases. This suggests a unique genetic predisposition affecting the immune response in children. Further research is needed to understand these differences and to tailor treatment strategies.

### 2.2. Clinical Features

The major clinical features of childhood rosacea are similar to those of adult rosacea. However, diagnostic criteria for the diagnosis of childhood rosacea have not been established. Various clinical aspects of childhood rosacea can be summarized as follows (Table 1).

**Figure 1:**
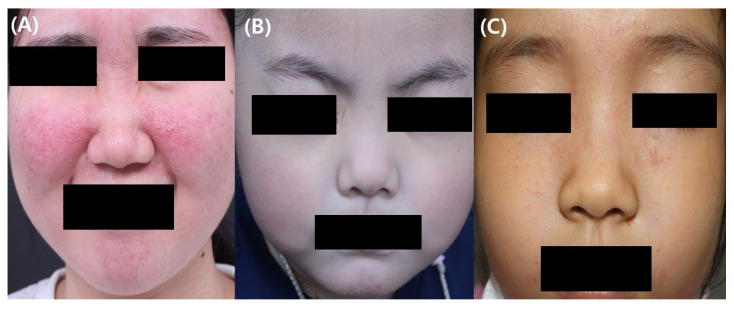
Representative clinical photographic images of childhood rosacea (**A**) Papulopustular rosacea in a 12-year-old girl. (**B**) Telangiectasia and erythema in a 4-year-old girl with rosacea (**C**) Granulomatous rosacea in an 8-year-old.

The papulopustular subtype is the most common form of childhood rosacea and is characterized by the appearance of papules and pustules on the face, especially on the central convex area, with or without persistent erythema or flushing [8] (Figure 1). Children may also complain of symptoms such as itching, burning, or stinging. In children, open or closed comedones can be observed as acne, and rosacea can coexist in some patients [17]. However, it is uncommonly observed. Some children initially manifest only flushing and develop papules and pustules later. 

In telangiectatic rosacea, the presence of persistent erythema with or without flushing is observed in pediatric patients. Flushing can be aggravated by potential triggers such as heat, exercise, and ultraviolet radiation and may persist for several minutes.

Granulomatous rosacea is characterized clinically by multiple flesh-colored papules and nodules on the face. There is usually less skin involvement in the periorbital area in granulomatous rosacea compared to in lupus miliaris disseminates faciei [18]. Granulomatous rosacea may resemble granulomatous perioral dermatitis clinically and histologically [19]. Controversy exists regarding whether pediatric granulomatous perioral dermatitis is a subtype of childhood rosacea or another disease entity. Some have suggested pediatric granulomatous perioral dermatitis to be one of the clinical manifestations of childhood rosacea [8], whereas others consider this to be a different entity [20]. Although additional studies are needed to further elucidate this association, granulomatous rosacea and pediatric granulomatous perioral dermatitis are now considered to be a spectrum of disease.

**Figure 2:**
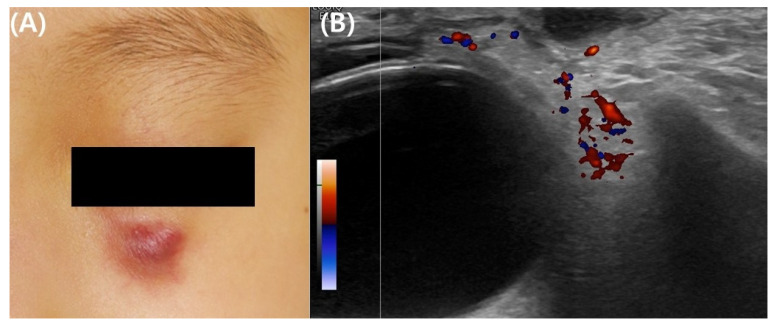
Clinical and ultrasonographic images of idiopathic facial aseptic granuloma in a 4-year-old girl. (**A**) Representative clinical photograph of idiopathic facial aseptic granuloma. (**B**) Ultrasonographic images show hypoechoic ovoid nodular lesion in infraorbital subcutis with relatively homogeneous boundaries and subtle vascularity in the surrounding areas.

Idiopathic facial aseptic granuloma (IFAG) is a single or small number of asymptomatic red to red-purple nodules on the cheeks (Figure 2). The histological findings are similar to those of granulomatous rosacea, and a previous report recommended that it should be regarded as a subtype of granulomatous rosacea as it has been observed in children with recurrent chalazion [21]. Biopsy and excision are often postponed because the condition is usually benign; there is a high likelihood of it resolving on its own [22]. Consequently, biopsy is rarely employed in diagnosing IFAG [22]. Instead, noninvasive methods such as ultrasonography and dermoscopy are frequently conducted in diagnosing IFAG. The ultrasonography findings in IFAG differ depending on the stage of progression of the lesion. Early lesions are observed as hypoechoic heterogeneous lesions with unclear borders and increased angiogenesis in the surrounding or internal areas. As IFAG progresses to the later stages, angiogenesis decreases, and it is characterized by a hypoechoic lesion with more homogeneous boundaries that are decreased [23]. Ultrasonography is useful for differentiating IFAG from other childhood rosacea differential diagnoses. Dermoscopy can be useful in diagnosing IFAG, with key dermoscopic features including an erythematous background, perifollicular hypopigmentation, follicular plugging, and nonbranching vessels [24]. 

An uncommon variant of rosacea, fulminant rosacea, has also been reported in some children. Fulminant rosacea is one of the severe subtypes of rosacea and is characterized by rapidly occurring erythematous papules, pustules, nodules, cysts, and tunnels. It is known to occur mainly in young women in their 20s and 30s [25], but it has also been reported to occur in people in their teens [25,26]. 

Ocular rosacea may be observed alone or in combination with other cutaneous manifestations. The symptoms corresponding to ocular rosacea include blepharitis, meibomian adenitis, recurrent stye, episcleritis, iritis, corneal neovascularization, keratitis, corneal ulcers and scars, marginal eyelid telangiectasia, and conjunctival congestion with or without subcorneal angiogenesis [27,28]. Pediatric patients frequently complain of ocular discomfort, foreign body sensation, or photophobia due to ocular rosacea [8]. In the case of ocular rosacea, signs of bilateral ocular involvement are more frequently observed than unilateral involvement [29].

In about 33–55% of childhood rosacea patients, orbital symptoms precede cutaneous involvement [8]. Therefore, children who complain of chronic orbital irritation need additional confirmation of skin invasion by rosacea or a family history of rosacea. Since pediatric ocular rosacea is a rare form of the disease, it may be underdiagnosed by ophthalmologists. Previous studies have reported that delays in the diagnosis of childhood ocular rosacea frequently occurred, with some delays of up to 7 years [27,28]. Early recognition is needed to prevent further complications and improve clinical outcomes, and a delayed diagnosis prevents the necessary systemic treatment of ocular rosacea [28]. 

### 2.3. Diagnosis of Childhood Rosacea

Diagnosis of childhood rosacea currently lacks clear diagnostic criteria and is primarily based on clinical features. Chamaillard et al. [6] suggested that childhood rosacea can be diagnosed when two or more of the following criteria are present: (1) facial flushing with recurrent or permanent erythema; (2) facial telangiectasia with no other causative disease; (3) papules and pustules without comedones; (4) preferential distribution of lesions on the convex areas of the face; or (5) ocular manifestations, including relapsing chalazion, ophthalmic hyperemia, or keratitis [6]. Generally, a biopsy is not required for diagnosing childhood rosacea, but it may be conducted to distinguish rosacea from other conditions. The histopathological findings can vary based on the clinical presentation of childhood rosacea, but dense dermal granulomatous inflammation in the perifollicular area is a common feature, similar to the cutaneous form of childhood rosacea [8]. Dermoscopy, a noninvasive tool initially used for skin tumors, is also effective in diagnosing the telangiectatic subtype of childhood rosacea, revealing a unique pattern of linear and polygonal vessels [30]. In adults with rosacea, especially the erythematotelangiectatic type, dermoscopy reveals vascular abnormalities such as polygonal and branched vessels. For papulopustular rosacea in adults, common findings include yellow dots representing dilated follicular infundibula filled with keratotic material and/or sebum, vascular polygons, and follicular scales [31]. Despite these characteristics, there is still a need for clear consensus guidelines from expert groups to establish diagnostic criteria for childhood rosacea.

### 2.4. Differential Diagnosis of Childhood Rosacea

The differential diagnoses of childhood rosacea include steroid rosacea, acne, sarcoidosis, and lupus erythematosus. Steroid rosacea is associated with a history of external steroid use, and most cases show an invasion of the skin around the mouth and nose. It is known to occur frequently when there is a family history of rosacea [32]. Acne, unlike childhood rosacea, usually has many comedones without flushing or telangiectasia. However, it should be kept in mind that childhood rosacea and acne may coexist in some preadolescent and adolescent patients.

Although rare in children, sarcoidosis can present as asymptomatic red-brown papules on the face. Childhood sarcoidosis is commonly observed in patients 9 to 15 years old [33]. Sarcoidosis in patients younger than 6 years is extremely rare and often presents with a triad of signs, including skin rash, uveitis, and arthritis without pulmonary involvement [33]. While systemic findings and laboratory evaluations can be useful in differentiating sarcoidosis from rosacea, it is important to note that such findings may often be absent in clinical practice, making the differentiation between these conditions more challenging. Patients with lupus erythematosus often have elevated antinuclear antibody titers [34,35]. A skin biopsy and an immunofluorescence study can help to clearly differentiate lupus erythematosus from rosacea [34]. 

### 2.5. Treatment of Childhood Rosacea

No specific guidelines for managing childhood rosacea have been suggested. Therefore, the treatment of childhood rosacea depends largely based on the treatment guidelines for adult rosacea. In general, it is necessary to identify and manage aggravating factors, such as exposure to environmental triggers, temperature change, emotional stress, and vigorous exercise [36]. In mild cases, topical therapies, such as metronidazole, azelaic acid, the combination of clindamycin/benzoyl peroxide, ivermectin, tacrolimus, and pimecrolimus, can be considered options for treating childhood rosacea. 

Metronidazole can be effective in treating rosacea as this agent has anti-inflammatory, antibiotic, antiparasitic, and antioxidative effects. The clinical efficacy of metronidazole in rosacea has been proven in various clinical trials [37,38] and a systematic review [39]. Although the previous clinical studies were conducted in adult populations, some case studies have reported the efficacy of topical metronidazole for treating childhood rosacea without severe side effects [21,30,40,41,42]. The common side effects associated with topical metronidazole include dry skin, burning, erythema, and pruritus. 

Azelaic acid gel exerts antibacterial, anti-inflammatory, and anti-keratinizing effects [43,44] and is usually used in managing rosacea and acne. It is effective in decreasing the papules and pustules of rosacea [45,46]. 

The combination of 1% topical clindamycin/5% benzoyl peroxide is also used for managing papulopustules in rosacea patients. Topical calcineurin inhibitors have been shown to be efficacious in treating rosacea due to their anti-inflammatory effects [44]. They are used for treating steroid-induced rosacea [47]. An open-label clinical trial of 1% pimecrolimus showed effects similar to those of 1% metronidazole cream in treating papulopustular rosacea with good tolerability [48]. However, topical calcineurin inhibitors, such as 1% pimecrolimus cream and 0.3% tacrolimus, are not indicated for use in children under the age of 2 years [49]. Topical ivermectin has anti-inflammatory and strong neurotoxic effects that are limited to nonvertebrate [44]. The double-blind placebo-controlled trials found that topical ivermectin was effective in treating adult papulopustular rosacea [50]. Although appropriate studies have not been performed regarding the safety of topical ivermectin in patients under 18 years, a case series by Noguera-Morel et al. [51] found that topical ivermectin was effective in treating papulopustular rosacea in children. Only transient and mild adverse events were observed [52]. 

Systemic oral treatment of childhood rosacea in combination with topical therapy is considered for moderate and severe childhood rosacea. As a systemic treatment, tetracycline-based antibiotics are effective for childhood rosacea, but the use of tetracycline-based antibiotics in children can cause permanent tooth discoloration and enamel dysplasia. Therefore, oral tetracycline-based antibiotics are not indicated for use in children under the age of 12 years. 

Erythromycin is considered a good treatment for children under 12 years old or those allergic to tetracyclines. Clarithromycin, azithromycin, and roxithromycin, which are second-generation macrolide antibiotics, have better bioavailability and fewer gastrointestinal side effects than erythromycin [36]. 

As systemic metronidazole is approved for use in individuals of all ages including infants and children for various infections, there are some case reports reporting the effectiveness of using systemic metronidazole in treating childhood rosacea [53,54]. 

Low-dose isotretinoin can also be considered a treatment option for patients with severe childhood rosacea. Isotretinoin is not recommended for use in children younger than 12 years of age, but it can be considered for patients with severe childhood rosacea who are refractory to treatment by appropriately monitoring serum lipid and liver enzyme levels [8]. In adolescent patients who have reached adult weight, the dose used for systemic treatment may follow the recommended dose for treating adults with rosacea. Laser or light-based treatments, such as pulsed-dye laser or intense pulse light, can also be used with local and systemic therapies to treat persistent erythema or vasodilation in managing childhood rosacea.

The treatment of IFAG typically involves a watchful, waiting approach due to its tendency to resolve spontaneously, often within an average of 12 months [55]. Unlike conventional rosacea, standard treatments such as topical or systemic antibiotics are generally ineffective for IFAG [55]. However, some cases have responded to oral or topical antibiotics [30,56,57,58]. In a recent retrospective study of 12 children with aseptic facial granuloma, treatment with oral macrolides (erythromycin or roxithromycin) led to lesion healing in an average of 5.25 months without any recurrences and was generally well tolerated, suggesting oral macrolides could be an effective treatment option for this condition [58]. Surgical excision is rarely necessary and reserved for a few cases [57].

For ocular rosacea, warm compresses and eyelid scrubbing have been recommended to improve eyelid hygiene [29]. Preservative-free artificial tears are also generally recommended. Topical antibiotics eyedrops, such as 1.5% topical azithromycin or 0.3% tobramycin eyedrops, can be used to control ocular inflammation and infection. Topical cyclosporin at 0.05% has been recommended for pediatric patients with prominent ocular surface inflammation who need longer topical steroid treatment [28,59]. Systemic antibiotics can also be prescribed for more severe forms of ocular rosacea. 

**Figure 3:**
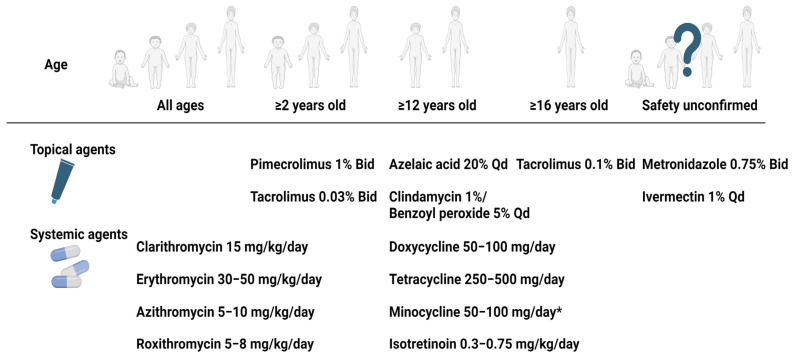
Treatment options for childhood rosacea according to age of the patient. * Minocycline is available ≥9 years old in some countries. Abbreviation: Bid, two times a day; Qd, once a day. Created with BioRender.com.

Due to age-related restrictions on medication use in childhood rosacea, we illustrate these considerations in Figure 3. Furthermore, as many clinical studies have not yet been conducted on pediatric populations, we compiled the clinical efficacy of the aforementioned medications in childhood rosacea patients in Table 2, summarizing the findings of single-agent effectiveness to date.

## 3. Conclusions

Although childhood rosacea is a relatively uncommon skin disease, it usually has a chronic disease course similar to that of adults and may show several clinical presentations (Table 3). There are no clear diagnostic guidelines for childhood rosacea. However, based on the clinical characteristics of childhood rosacea, which include papulopustular, telangiectasia, granulomatous, IFAG, and ocular rosacea, and excluding other differential diagnoses, a diagnosis of childhood rosacea can be confirmed. As there is the potential for underdiagnosing childhood rosacea, there is a need for special awareness of childhood rosacea in the clinical setting. 

Early and appropriate treatment of childhood rosacea patients will be helpful in managing childhood rosacea. Of note, as ocular rosacea alone can precede the skin signs and symptoms of cutaneous rosacea, consultation with ophthalmologist is important for early diagnosing and proper management of patients with childhood rosacea. Moreover, as having rosacea during childhood may increase the risk of developing rosacea as an adult [68], close and regular follow-up for childhood rosacea should be conducted even after the clinical remission of the rosacea symptoms.

## Figures and Tables

**Table 1:** Representative clinical manifestations of childhood rosacea.

Clinical Manifestation	Characteristics
Papulopustular	Crops of papules and pustules with or without facial erythema or flushing
Telangiectatic	Persistent erythema with or without flushing
Granulomatous	Firm erythematous papules and pustules on a background of a normal-appearing skin
Idiopathic facial aseptic granuloma	Non-tender solitary or multiple red to violaceous nodules on the cheeks
Ocular rosacea	Occurs with or without cutaneous manifestations ofBlepharoconjuctivitis, meibomitis, recurrent chalazion, episcleritis, iritis, corneal vascularization, keratitis, corneal ulcer and scarring, lid margin telangiectasia, conjunctival hyperemia with or without inferior corneal vascularization

**Table 2:** Summary on the effectiveness of a single therapeutic agent in management of childhood rosacea.

Reference	Age (Number of Patients)	Subtype	Doses and Duration	Clinical Response
*Azithromycin alone*				
Zanella et al. [60]	3 y (*n* = 1)	IFAG	1.5% 3 days in a row every 15 days for 3 months	Favorable outcome
Doan et al. [61]	4–16 y (*n* = 16)	O	1.5% Bid for 3 days every 10 days	Effective in 15 of 16 patients
*Clarithromycin alone*				
Borok et al. [30]	1 y (*n* = 1)	IFAG	15 mg/kg BID for 4 months	Complete resolution
Neri et al. [21]	4 y (*n* = 1)	G	15 mg/kg BID for 2 months	Complete resolution
*Doxycycline alone*				
Donaldson et al. [62]	Mean 9.2 y (*n* = 2)	O	50 mg or 100 mg BID	Well tolerated
Leoni et al. [54]	14 y (*n* = 1)	G	100 mg daily for 2 months	Complete remission
Leoni et al. [54]	12 y (*n* = 2)	O	100 mg daily	Complete remission
*Erythromycin alone*				
Gonser et al. [63]	2 y (*n* = 1)	PP and O	300 mg BID for 10 months	Complete remission
Neri et al. [21]	2 y (*n* = 1)	G	50 mg/kg TID for 2 months	Almost complete remission
*Isotretinoin alone*				
Cantarutti et al. [64]	10 y (*n* = 1)	G	0.5 mg/kg daily for 6 months	Almost complete disappearance, worsened after tapering
Lee and Fischer [65]	2–7 y (*n* = 4)	IFAG	0.25 mg/kg daily for 6–9 months	Successful treatment, minimal side effects
Sanchez-Espino and Sibbald [66]	7 y (*n* = 1)	IFAG	1 mg/kg twice weekly	Clear resolution
*Ivermectin alone*				
Brown et al. [67]	12 y (*n* = 1)	PP and O	A single dose (250 μg/kg)	Significant improvement at 1 month
*Metronidazole alone*				
Borok et al. [30]	2 y (*n* = 1)	IFAG	0.75% BID for 4 months	Complete resolution at follow-up
Eghlileb and Finlay [41]	16 y (*n* = 1)	G	0.75% BID for 2 months	Some improvement
Galindo-Ferreiro et al. [42]	3–10 y (*n* = 1)	IFAG	0.75% BID	Partial improvement
Garais et al. [53]	1 y (*n* = 1)	IFAG	20 mg/kg daily for 2 months	Complete resolution
Leoni et al. [54]	1–5 y (*n* = 10)	PP; PP and O; ETR, PP and O; PP and O; and O	20–30 mg/kg per day for at least 3 months	Alternative treatment for ocular and cutaneous rosacea

Abbreviations: BID, twice a day; ETR, erythematotelangiectatic; G, granulomatous; IFAG, idiopathic facial aseptic granuloma; O, ocular; PP, papulopustular; TID, three times a day; y, years.

**Table 3:** Comparison of childhood and adult rosacea.

Feature	Childhood Rosacea	Adult Rosacea
Age of onset	4–5 years old	35 to 45 years in women 45 to 55 years in men
Sex	Similarly observed both in boys and girls	Predominance in women
Clinical presentation	-Papulopustular rosacea -Telangiectatic rosacea -Granulomatous rosacea -IFAG: pediatric specific subtype -Ocular rosacea: more common and more frequently preceding cutaneous features in children -Lack of consensus on the classification for childhood rosacea	-Major subtypes Papulopustular Erythematotelangiectatic Phymatous Ocular rosacea -Other subtypes Granulomatous rosacea Neurogenic rosacea
Treatment	-Lack of consensus, refer to the adults’ treatments	-General skin care, topical therapy, systemic therapy, laser therapy or IPL or surgical intervention can be conducted based on the patient’s symptom.
Prognosis	-Having rosacea during childhood may increase the risk of developing rosacea as an adult -IFAG: resolve spontaneously, often within an average of 12 months	Chronic conditions with fluctuating course

Abbreviation: IFAG, idiopathic facial aseptic granuloma; IPL, intense pulsed light.

## Data Availability

Not applicable.
